# Supplementary material for: Cas9 targeted nanopore sequencing with enhanced variant calling improves CYP2D6-CYP2D7 hybrid allele genotyping
Source: PLoS Genet. 2022 Sep 23;18(9):e1010176. doi: 10.1371/journal.pgen.1010176 (PMC9534437; doi:10.1371/journal.pgen.1010176)
Supplement: S1 Fig — The x-axis represents the DNA fragment size (non-linear scale). The y-axis represents the fluorescent signal proportional to the amount of DNA. A: NA12878, B:HG01190, and C:GM19785. (PDF) [file pgen.1010176.s001.pdf]

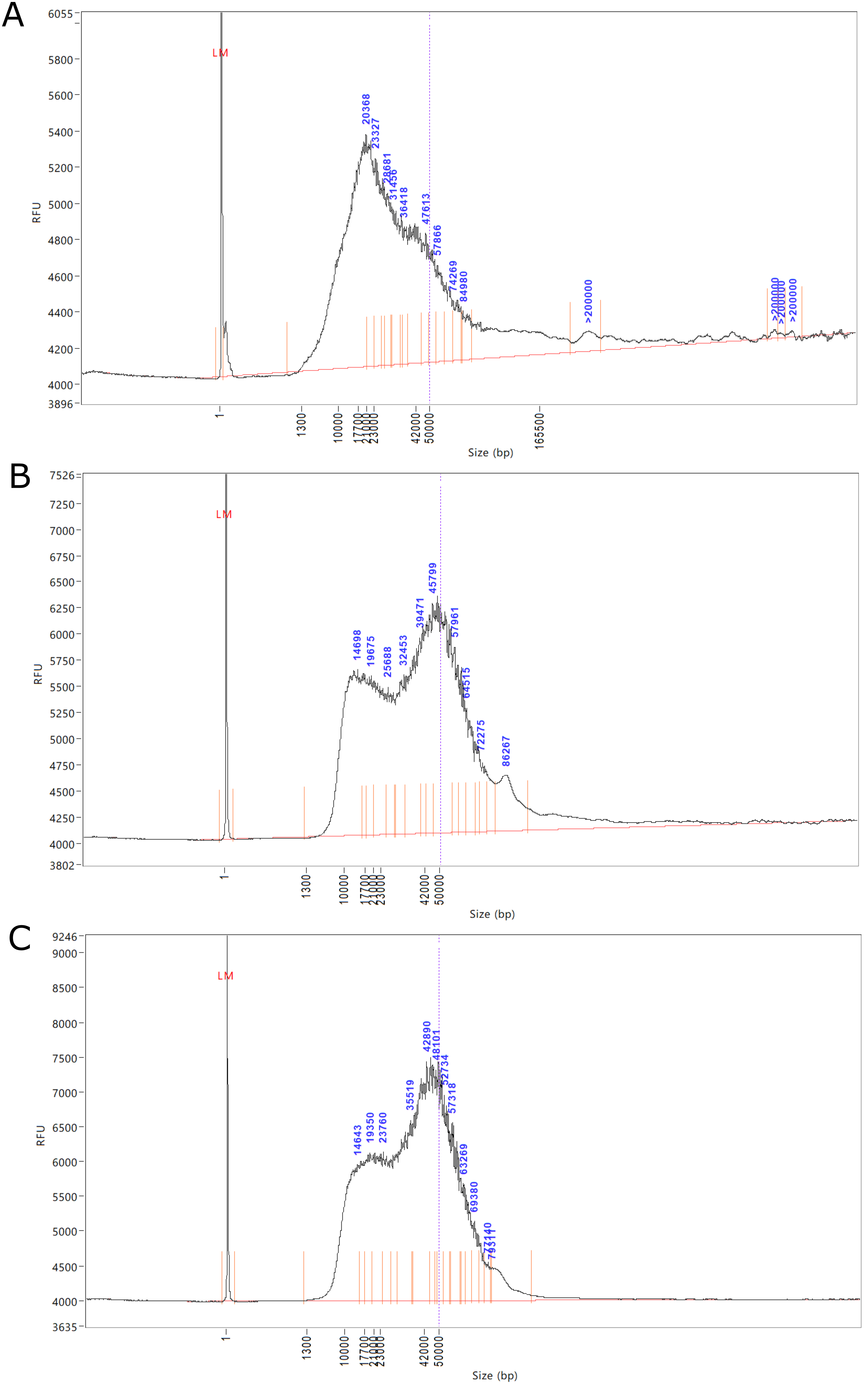

S1 Fig Femto pulse profiles of the used DNA samples. The x-axis represents the DNA fragment size (non-linear scale). The y-axis represents the fluorescent signal proportional to the amount of DNA. A: NA12878, B: HG01190, and C: GM19785.
