# Supplementary material for: Cas9 targeted nanopore sequencing with enhanced variant calling improves CYP2D6-CYP2D7 hybrid allele genotyping
Source: PLoS Genet. 2022 Sep 23;18(9):e1010176. doi: 10.1371/journal.pgen.1010176 (PMC9534437; doi:10.1371/journal.pgen.1010176)
Supplement: S2 Fig — The positions of the gRNAs are indicated with vertical lines and the sequencing direction is indicated with arrows on top of the vertical lines. Reads are split by allele. The position where gRNA9 binds off-target is zoomed in. This recognition site shows one mismatch (red) and one mutation (green). (PDF) [file pgen.1010176.s002.pdf]

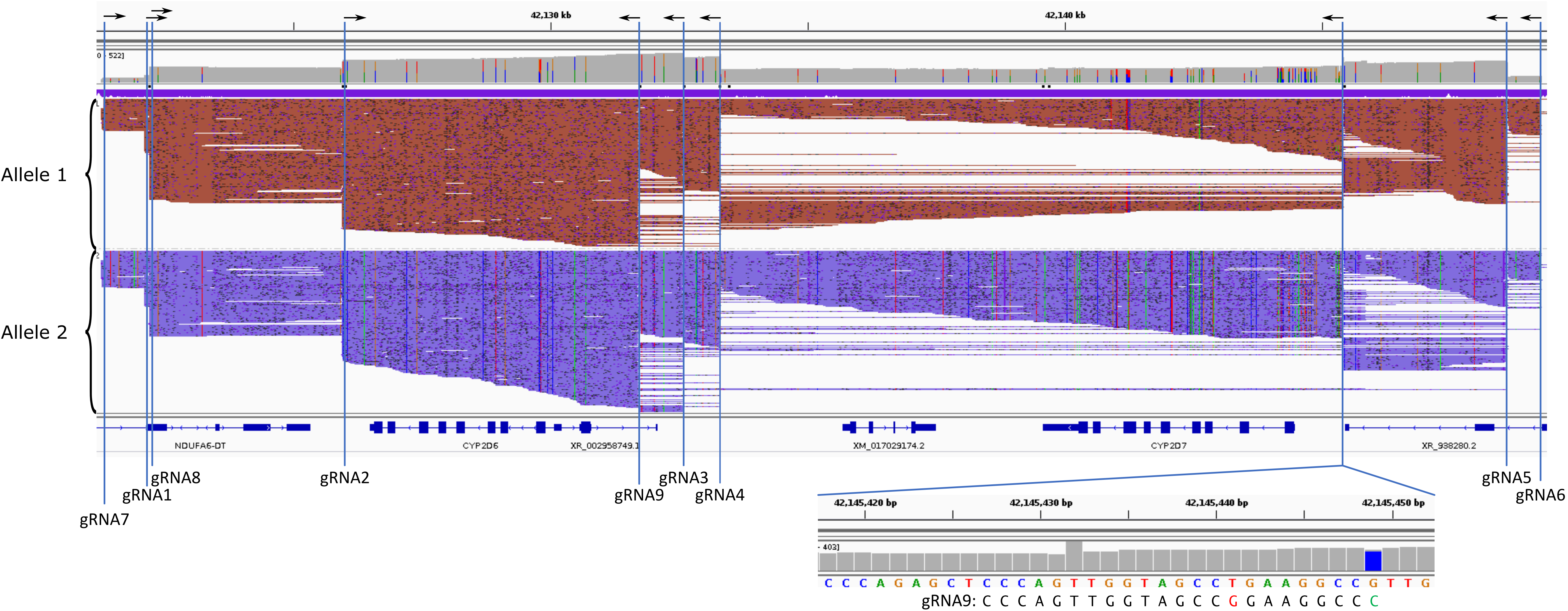


*S2 Fig* Reads of the NA12878 DNA, sequenced on a MinION flow cell, mapped on the GRCh38 reference genome. The positions of the gRNAs are indicated with vertical lines and the sequencing direction is indicated with arrows on top of the vertical lines. Reads are split by allele. The position where gRNA9 binds off-target is zoomed in. This recognition site shows one mismatch (red) and one mutation (green).
