# Supplementary material for: Cas9 targeted nanopore sequencing with enhanced variant calling improves CYP2D6-CYP2D7 hybrid allele genotyping
Source: PLoS Genet. 2022 Sep 23;18(9):e1010176. doi: 10.1371/journal.pgen.1010176 (PMC9534437; doi:10.1371/journal.pgen.1010176)
Supplement: S3 Fig — The positions of the gRNAs are indicated with vertical lines and the sequencing direction is indicated with arrows on top of the vertical lines. gRNA3 cut reads generated by gRNA4, causing a lower depth on CYP2D6. (PDF) [file pgen.1010176.s003.pdf]

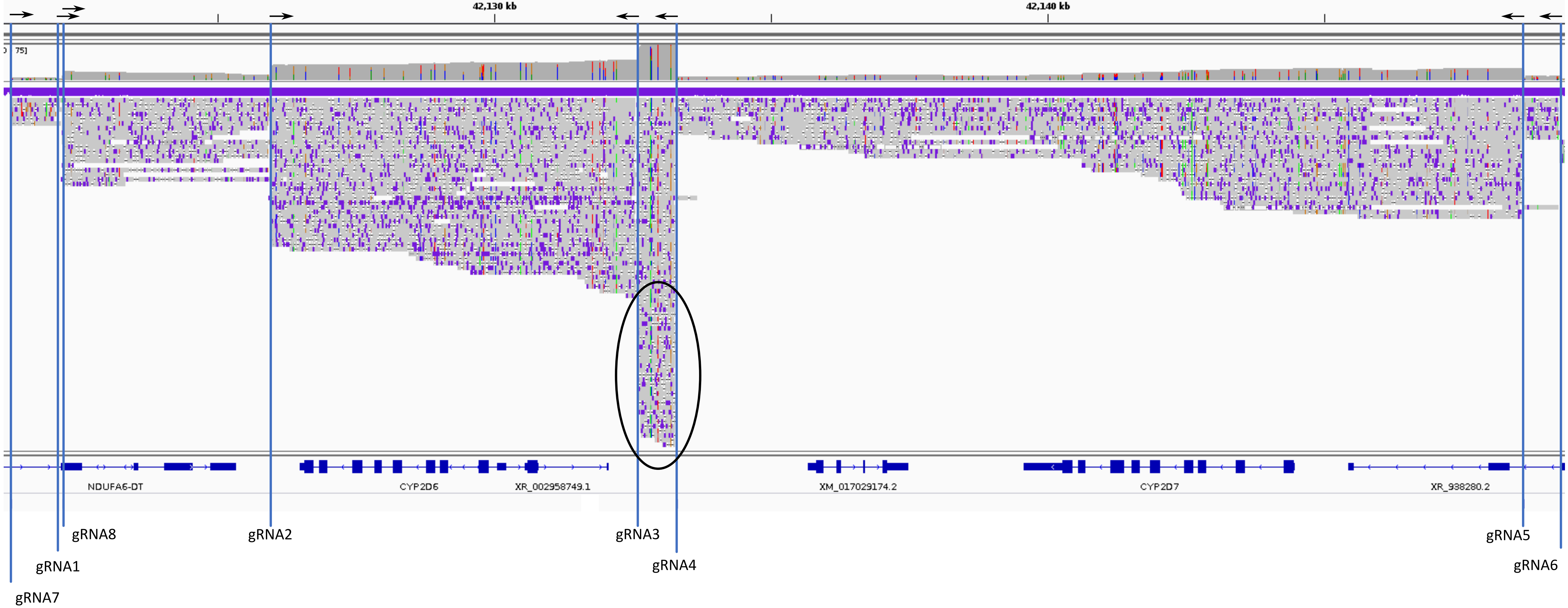


*S3 Fig* Reads of the NA12878 DNA, sequenced on a Flongle flow cell, mapped on the GRCh38 reference genome. The positions of the gRNAs are indicated with vertica l lines and the sequencing direction is indicated with arrows on top of the vertical lines. gRNA3 cut reads generated by gRNA4, causing a lower depth on *CYP2D6*.
