# Supplementary material for: Cas9 targeted nanopore sequencing with enhanced variant calling improves CYP2D6-CYP2D7 hybrid allele genotyping
Source: PLoS Genet. 2022 Sep 23;18(9):e1010176. doi: 10.1371/journal.pgen.1010176 (PMC9534437; doi:10.1371/journal.pgen.1010176)
Supplement: S4 Fig — The HGcombined dataset was used to generate this figure, which is the dataset containing both the positively selected reads from the AS pores and all the reads from the conventionally sequencing pores. The positions of the gRNAs are indicated with vertical lines and the sequencing direction is indicated with arrows on top of the vertical lines. Reads are split by allele, and gray reads are clipping ends that were cut in-silico and mapped separately. The cut position of the clipping ends indicating the start and end position of the deletion and insertion are indicated in gray. (PDF) [file pgen.1010176.s004.pdf]

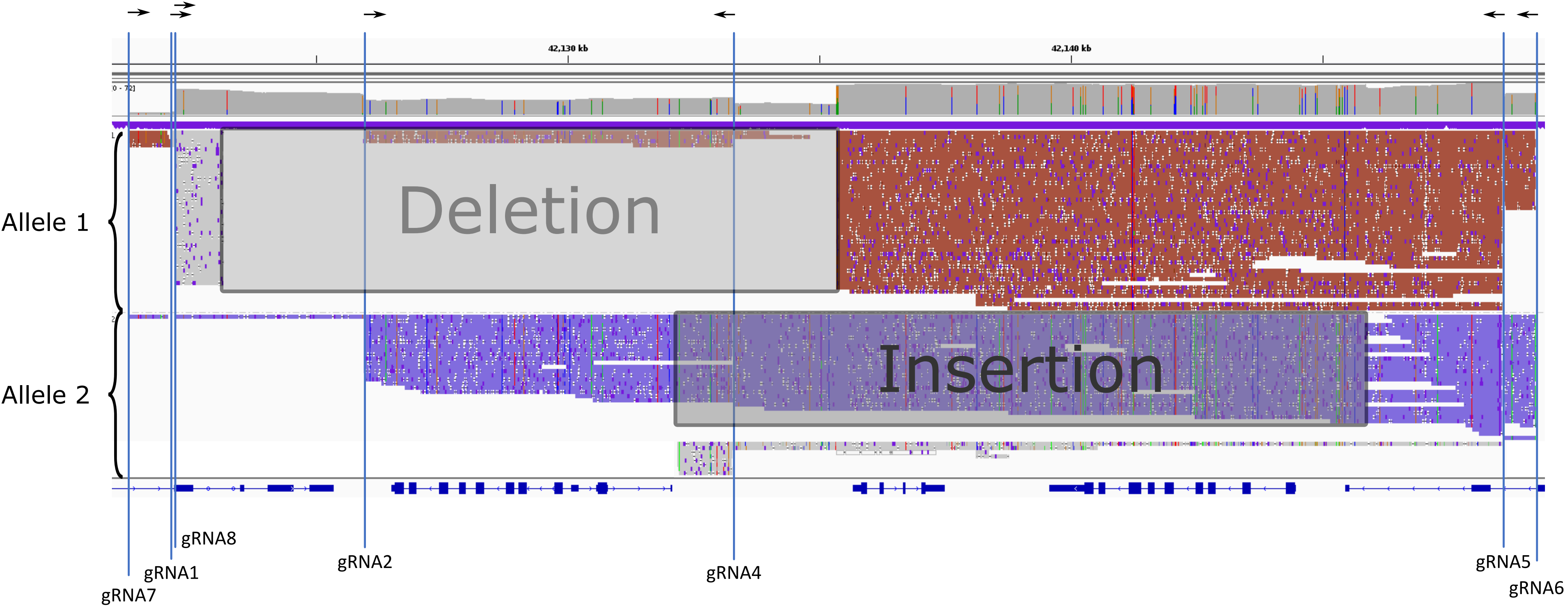

S4 Fig Reads of the HG01190 DNA, sequenced on a MinION flow cell, mapped on the GRCh38 reference genome. The $HG_{combined}$ dataset was used to generate this figure, which is the dataset containing both the positively selected reads from the AS pores and all the reads from the conventionally sequencing pores. The positions of the gRNAs are indicated with vertical lines and the sequencing direction is indicated with arrows on top of the vertical lines. Reads are split by allele, and gray reads are clipping ends that were cut in-silico and mapped separately. The cut position of the clipping ends indicating the start and end position of the deletion and insertion are indicated in gray.
