# Supplementary material for: Cas9 targeted nanopore sequencing with enhanced variant calling improves CYP2D6-CYP2D7 hybrid allele genotyping
Source: PLoS Genet. 2022 Sep 23;18(9):e1010176. doi: 10.1371/journal.pgen.1010176 (PMC9534437; doi:10.1371/journal.pgen.1010176)
Supplement: S5 Fig — The GMcombined dataset was used to generate this figure, which is the dataset containing both the positively selected reads from the AS pores and all the reads from the conventionally sequencing pores. The positions of the gRNAs are indicated with vertical lines and the sequencing direction is indicated with arrows on top of the vertical lines. Reads are split by allele. gRNA2* indicates the position where gRNA2 cuts before the CYP2D6-CYP2D7 *13 hybrid. The reads of the hybrid map on the CYP2D7 gene, but the first part of the hybrid originates from the CYP2D6 gene, resulting in a visual gap in the alignment. (PDF) [file pgen.1010176.s005.pdf]

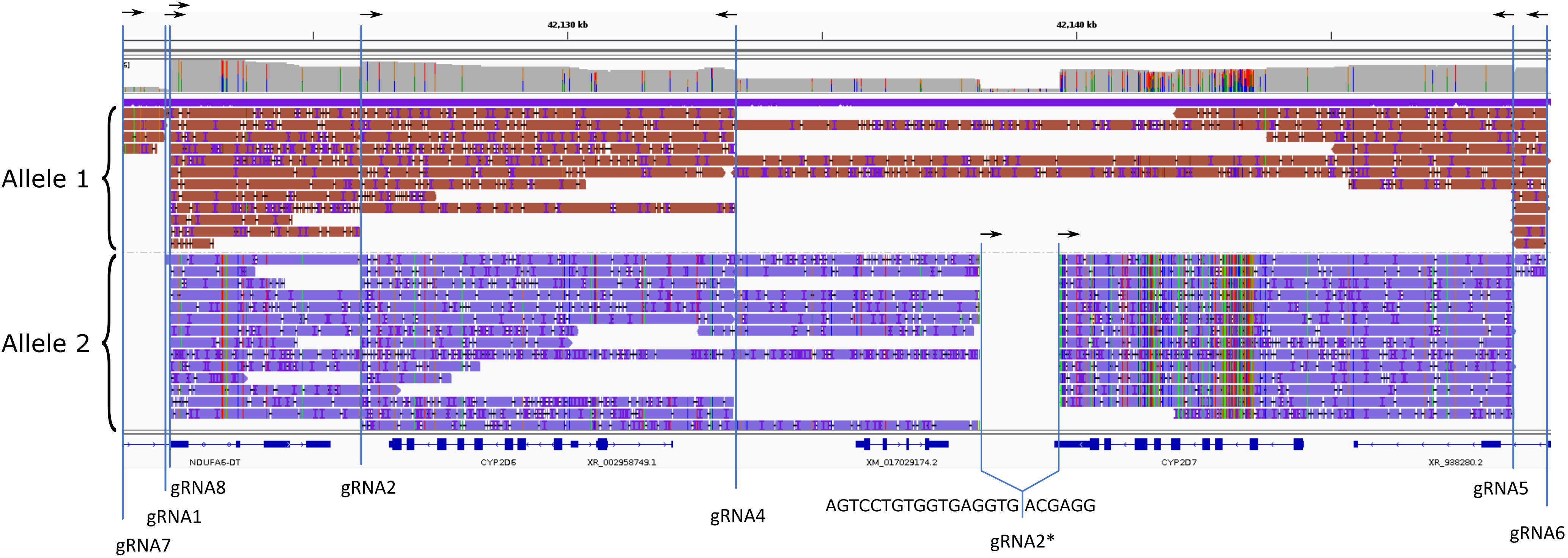

S5 Fig Reads of the GM19785 DNA, sequenced on a MinION flow cell, mapped on the GRCh38 reference genome. The $GM_{combined}$ dataset was used to generate this figure, which is the dataset containing both the positively selected reads from the AS pores and all the reads from the conventionally sequencing pores. The positions of the gRNAs are indicated with vertical lines and the sequencing direction is indicated with arrows on top of the vertical lines. Reads are split by allele. gRNA2* indicates the position where gRNA2 cuts before the *CYP2D6-CYP2D7* *13 hybrid. The reads of the hybrid map on the *CYP2D7* gene, but the first part of the hybrid originates from the *CYP2D6* gene, resulting in a visual gap in the alignment.
