# Supplementary material for: Cas9 targeted nanopore sequencing with enhanced variant calling improves CYP2D6-CYP2D7 hybrid allele genotyping
Source: PLoS Genet. 2022 Sep 23;18(9):e1010176. doi: 10.1371/journal.pgen.1010176 (PMC9534437; doi:10.1371/journal.pgen.1010176)
Supplement: S1 Text — Table A: Overview of the used guide RNAs (gRNAs). Table B: Comparison of small SNV and INDEL variant detection of the Medaka Variant pipeline and the new CoLoRGen tool in the NA12878 DNA sample. Reference: Krusche et al. [36]. Table C: Comparison of structural variant detection of different state-of-the-art structural variant tools and the new CoLoRGen tool in the NA12878, HG01190 and GM19785 DNA samples. For each tool the number of deletions and insertions are given. Between parentheses the length of each variant is given. Green: correctly detected structural variant; red: incorrectly detected structural variant; orange: multiple overlapping structural variants are detected although only one variant is present in the reference. Reference: Get-RM studies [15,16]. †: the found regions show overlap. (DOCX) [file pgen.1010176.s007.docx]

Supporting Information: Cas9 targeted nanopore sequencing with enhanced variant calling improves *CYP2D6-CYP2D7* hybrid allele genotyping

# Tables

Table A Overview of the used guide RNAs (gRNAs).

| gRNA | Sequence | PAM | Efficiency | Coordinates | Postion relative to *CYP2D6* and *CYP2D7* |
| --- | --- | --- | --- | --- | --- |
| gRNA1 | CCATTCACCCTTATGCTCAG | GGG | 64.42 | 42122120  (+) | Upstream |
| gRNA2 | AGTCCTGTGGTGAGGTGACG | AGG | 63.96 | 42125967  (+) | Upstream |
| gRNA3 | GCCATACAATCCACCTGTAG | AGG | 57.87 | 42132600  (-) | Between *CYP2D6* and *CYP2D7* |
| gRNA4 | CTTTCCGACATACACGCAAT | GGG | 56.42 | 42133313  (-) | Between *CYP2D6* and *CYP2D7* |
| gRNA5 | TTCCCCACTTTTTACTACAC | AGG | 50.30 | 42148614  (-) | Downstream |
| gRNA6 | CAAAGTCCATGCGTAAGTCT | TGG | 50.42 | 42149274  (-) | Downstream |
| gRNA7 | TCTCACCAGCAATAACCGAG | AGG | 75.07 | 42121282  (+) | Upstream |
| gRNA8 | ACCTCCGGTTGCTTCCTGAG | GGG | 69.88 | 42122214  (+) | Upstream |
| gRNA9 | GGGCCTTCCGGCTACCAACT | GGG | 51.62 | 42131731 (-) | Between *CYP2D6* and *CYP2D7* |

Table B Comparison of small SNV and INDEL variant detection of the Medaka Variant pipeline and the new CoLoRGen tool in the NA12878 DNA sample. Reference: Krusche et al. (1).

| Run | Correctly called and phased SNVs (*CYP2D6 + CYP2D7*) | Incorrectly called SNVs  (*CYP2D6 + CYP2D7*) | Correctly called and phased INDELs  (*CYP2D6 + CYP2D7*) | Incorrectly INDELs  (*CYP2D6 + CYP2D7*) |
| --- | --- | --- | --- | --- |
| Reference | 11 + 26 | / | 1 + 1 | / |
| CoLoRGen | 11 + 26 | 2 + 5 | 1 + 0 | 0 + 4 |
| Medaka | 11 + 26 | 2 + 6 | 1 + 1 | 1 + 3 |

Table C Comparison of structural variant detection of different state-of-the-art structural variant tools and the new CoLoRGen tool in the NA12878, HG01190 and GM19785 DNA samples. For each tool the number of deletions and insertions are given. Between parentheses the length of each variant is given. Green: correctly detected structural variant; red: incorrectly detected structural variant; orange: multiple overlapping structural variants are detected although only one variant is present in the reference. Reference: Get-RM studies (2,3). †: the found regions show overlap.

|  | NA12878 | | HG01190 | | GM19785 | |
| --- | --- | --- | --- | --- | --- | --- |
|  | deletion | insertion | deletion | insertion | deletion | insertion |
| Reference | / | *68 | *5 | *68 | / | / |
| CoLoRGen | / | 1 (13,680 bp) | 1 (12,152 bp) | 1 (13,680 bp) | / | / |
| NanoVar (PASS) | / | / | / | 1 (13,838 bp) | / | / |
| Sniffles (PASS) | 2 (12,282 bp, 12,152 bp) † | 3 (12,154 bp, 13,708 bp, 13,659 bp) † | 2 (12,454 bp, 12,155 bp) † | 1 (1,006 bp) | 1 (13,656 bp) | / |
| SVIM (QUAL >=3, PASS) | / | 2 (13,638 bp, 13,613 bp) † | / | 1 (13,424 bp) | 2 (13,696 bp, 13,663 bp) † | / |

Reference

1. Krusche P, Trigg L, Boutros PC, Mason CE, De La Vega FM, Moore BL, et al. Best practices for benchmarking germline small-variant calls in human genomes. Nat Biotechnol. 2019 May 11;37(5):555–60.

2. Pratt VM, Everts RE, Aggarwal P, Beyer BN, Broeckel U, Epstein-Baak R, et al. Characterization of 137 Genomic DNA Reference Materials for 28 Pharmacogenetic Genes: A GeT-RM Collaborative Project. J Mol Diagnostics. 2016 Jan 1;18(1):109–23.

3. Gaedigk A, Turner A, Everts RE, Scott SA, Aggarwal P, Broeckel U, et al. Characterization of Reference Materials for Genetic Testing of CYP2D6 Alleles: A GeT-RM Collaborative Project. J Mol Diagnostics. 2019 Nov 1;21(6):1034–52.
