# Supplementary material for: Cas9 targeted nanopore sequencing with enhanced variant calling improves CYP2D6-CYP2D7 hybrid allele genotyping
Source: PLoS Genet. 2022 Sep 23;18(9):e1010176. doi: 10.1371/journal.pgen.1010176 (PMC9534437; doi:10.1371/journal.pgen.1010176)
Supplement: S6 Fig — The three deletions were located in homopolymeric regions and the SNV is a silent mutation. (PDF) [file pgen.1010176.s006.pdf]

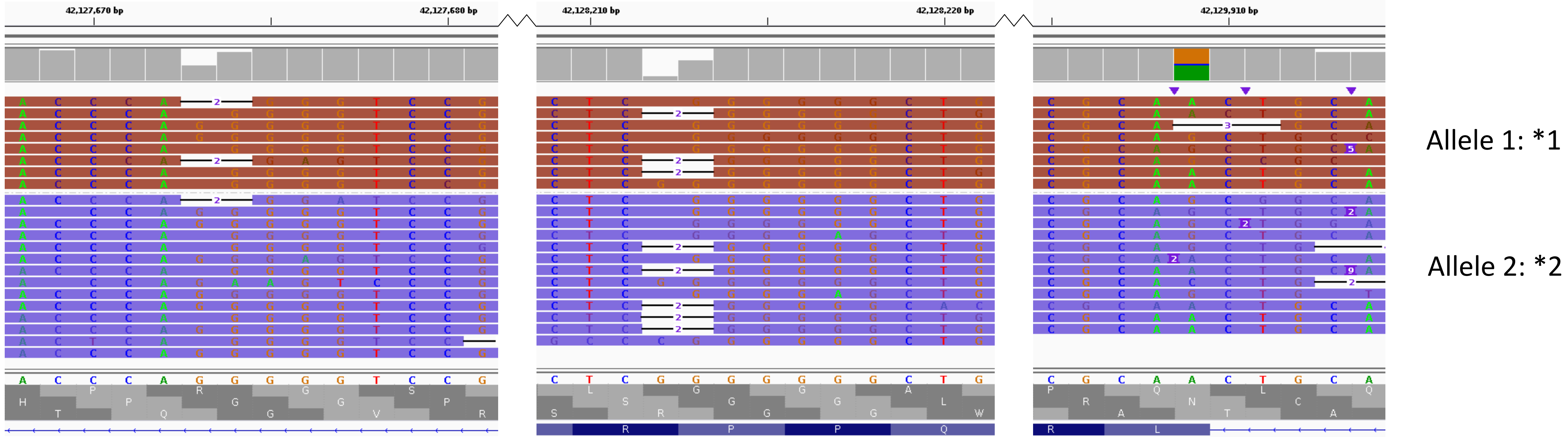

S6 Fig CoLoRGen detected four additional small variants in the GM19785 cell line that are not present in the sub-allele definitions. The three deletions were located in homopolymeric regions and the SNV is a silent mutation.
